# Kinetics of Ordering and Decomposition in Ti-Al-X (X = Si, Zr) Alloys: Monte Carlo Modeling

**DOI:** 10.3390/ma15165722

**Published:** 2022-08-19

**Authors:** Mikhail Petrik, Ilya Razumov, Yuri Gornostyrev, Inna Naschetnikova, Artemyi Popov

**Affiliations:** 1 Mikheev Institute of Metal Physics, Ural Branch, Russian Academy of Sciences, Ekaterinburg 620108, Russia; 2 Heat Treatment and Physics of Metals Department, Ural Federal University Named after The First President of Russia B.N. Yeltsin, Ekaterinburg 620002, Russia

**Keywords:** titanium alloys, ordering and decomposition kinetics, ab initio calculations, Monte Carlo modeling

## Abstract

To investigate the ordering and decomposition processes in Ti-Al-X [X = Si, Zr] alloys, the Monte Carlo simulations with first-principles parametrization of interatomic interactions were employed. It was shown that the processes of ordering and the precipitation in the Ti-Al system are closely related, and the stage of homogeneous ordering precedes the formation of ordered Ti_3_Al particles. It was found that the duration of homogeneous ordering is very sensitive to the annealing temperature and composition of alloy, and that precipitation becomes preferable as the temperature rises. In particular, uniform ordering of alloy Ti-12 at % Al was found during long-term holding at temperature below 850 K, while annealing at 1000 K resulted in formation of ordered Ti_3_Al particles. The obtained results agree well with the experimental data and allow explaining the features of the microstructure formed during annealing of the Ti-Al-X alloys.

## 1. Introduction

Titanium-aluminum alloys are distinguished by low density, high specific strength and good high-temperature properties that make them attractive for many important applications [1,2,3], especially in the aerospace industry. The Ti-rich edge of the Ti-Al equilibrium phase diagram displays an extensive primary solid solution with hexagonal structure (α phase) [4]. Between approximately 11% and 22at%Al, the two-phase field is stable up to a temperature of 1180 °C. Short range order formation is also observed [5,6] in a wide range of concentrations before the α + α_2_ two-phase region. The α_2_ phase (ordered DO_19_ structure based upon the intermetallic Ti_3_Al) plays an essential role in the mechanical behavior of Ti-Al alloys. The formation of ordered phase improves the strength but decreases the ductility of the alloy [7,8]. Therefore, a theoretical understanding of the mechanism of ordering in the Ti-Al alloys is an important step to control their structure and properties.

The effect of heat treatment on α_2_ precipitation in Ti-Al alloys with different concentrations of Al and other alloying elements has been studied in papers [5,8,9,10,11,12,13]. The decomposition mechanism of Ti-rich Ti-Al alloys may be considered as a ‘conditional spinodal’ [14,15] when superlattice reflections appear in diffraction patterns before any notable composition variations [9,11,16,17]. As was shown in [16], the strength of the superlattice reflections in Ti-15at%Al increases with both aging time and temperature, showing the development of order in the alloy prior to the chemical phase separation. The decomposition in Ti-15at%Al develops faster when the temperature of annealing increases from 550 °C to 750 °C [17]. Wherein, ordered regions appear to be spherical in shape initially and then elongate to form ellipsoids after longer ageing times [8,17] and remain rather small (up to 150 × 30 nm for the considered composition).

Thus, the ordering and decomposition processes are competitive during heat treatment of the Ti-Al alloy, and significant ordering can occur in the alloy prior to the onset of chemical phase separation. As a result, various structural states can be realized depending on the annealing temperature and alloy composition [6,12,17,18,19,20,21,22,23]. Homogeneous ordering has been observed in alloys containing 12.5 at% Al at annealing temperatures up to 600 °C, and the formation of ordered particles of Ti_3_Al was found at 700 °C [19,22]. It was shown in [21] that long ageing times at 500 °C lead to the formation of Ti_3_Al precipitates, while reduced times result in short range ordering.

As follows from the available experimental results, the ordering mechanism of near-α Ti-Al alloy is complex and very sensitive to temperature and composition. However, microscopic reasons for this behavior remain a subject of debate. Moreover, it is unclear whether a (quasi)equilibrium state is reached (as discussed in [18]), or the observed features are a consequence of the kinetics of the decomposition process [16]. There is only one study [16] where Monte Carlo simulations have been used to demonstrate the change from ordering to precipitation kinetics with increasing time.

To clarify the interplay of the ordering and decomposition processes in Ti-Al-X (X = Si, Zr) alloys, we carried out the ab initio based atomistic simulations at various temperatures and alloy compositions. We showed, in agreement with previous calculations [16], that switching of ordering and decomposition processes during annealing has a kinetic nature. The duration of these stages was found to be very sensitive to the annealing temperature and composition of the alloy. This opens a way to control the structural state and properties of considered alloys.

## 2. Materials and Methods

Calculations of the energy of Ti-(AlX) [X = Zr, Si] alloys were performed by the density functional theory (DFT) method in the plane augmented wave (PAW) approximation [24] implemented in Vienna Ab initio Simulation Package (VASP) software package [25]. The exchange-correlation effects were considered in the generalized gradient approximation (GGA) with the Perdew–Burke–Ernzerhof (PBE) parametrization [26], which provides high accuracy in calculating the total energies of alloys. For DFT calculations, 128 atomic supercells containing 4 × 4 × 4 hcp titanium cells were used. Sampling of the first Brillouin zone was performed using a k-grid of 4 × 4 × 4 k-points built according to the Monkhorst-Pack scheme [27]. The calculated lattice parameters a_0_(Ti) = 2.95 Å, c_0_(Ti) = 4.69 Å agree well with the experimental data at room temperature.

The dissolution energy of *X* atom in the *Ti* matrix was calculated using the following formula (see for example [28]):

(1) $$E_{sol}=E_{tot}\left[Ti_{N}X_{1}\right]-NE_{at}\left[Ti\right]-E_{at}\left[X\right]$$

where $E_{tot}\left[Ti_{N}X_{1}\right]$ is the total energy of a cell containing *N* atoms of Ti and one alloying element atom X = Al, Zr, Si; $E_{at}\left[Ti\right]$ and $E_{at}\left[X\right]$ are the energies per atom in the hcp *Ti* lattice and in the lattice corresponding to the ground state of element *X*. The energies of effective pair interaction between atoms of alloying elements in the titanium lattice were calculated as [28]:

(2) $$E_{int}^{\left(n\right)}=E_{tot}^{\left(n\right)}\left[Ti_{N-2}X_{2}\right]-2E_{tot}\left[Ti_{N}X_{1}\right]-128E_{at}\left[Ti\right]$$

where *n* is the number of the coordination sphere; $E_{tot}^{\left(n\right)}\left[Ti_{N-2}X_{2}\right]\;$ is the total energy of the alloy containing two atoms of *X* at a distance from each other corresponding to the *n*-th coordination sphere.

To consider the effect of thermal expansion on the dissolution and interaction energies, the calculation was carried out for lattice parameter Ti corresponding to two temperatures: T_1_ = 298 K and T_2_ = 1000 K. In this case, the lattice parameters *a* and *c* were determined according to the relations *a* = *a*_0_(1 + α∆T), *c* = *c*_0_(1 + β∆T), where *a*_0_ = 2.95 Å, *c*_0_ = 4.69 Å are lattice parameters at room temperature, α and β are linear thermal expansion coefficients, α = $17.6\times 10^{-6}$ $K^{-1}$; β = $5\times 10^{-6}$ $K^{-1}$ [29], ∆T is the change in temperature relative to room temperature. When performing total energy calculations with a = 2.99 Å, c = 4.71 Å (corresponding to T = 1000 K), the cell volume and shape were fixed, whereas the relaxation of the positions of atoms inside the supercell was carried out.

Modeling of ordering and decomposition processes in binary (Ti-Al) and ternary alloys Ti-Al-Zr, Ti-Al-Si was carried out by the kinetic Monte Carlo method with direct exchange of the nearest atoms [30]. For this purpose, a crystallite with a size of 50 × 50 × 50 elementary cells of hcp titanium was used. At each step of the algorithm, a pair of the nearest atoms of different types was selected. In binary Ti-Al alloy, the probability of swapping a pair of atoms was fully determined by the difference in the energies of the initial and final configurations according to the Metropolis rule. When calculating the energies of the initial and final configurations, the effective pair interactions between the atoms of alloying elements up to the sixth coordination sphere computed by the PAW-VASP method were used.

In a ternary alloy, the swapping probability depends on the types of atoms in the pair under consideration. It was assumed that for a solute atom with a higher diffusion mobility, the swapping with the matrix atom occurs with the Metropolis probability, as in a binary alloy. For a solute atom with a lower diffusion mobility, its exchange with a matrix atom is carried out with the Metropolis probability multiplied by the ratio of the corresponding diffusion coefficients of low- and high-mobile atoms [31,32]. The diffusion of Si in the α-Ti matrix is several orders of magnitude faster than the diffusion of Al [32]; therefore, to reduce the time of calculation and obtain qualitative conclusions, it was assumed that the first diffusion is only 10 times greater than the second one. It was also assumed that for a pair of Al and Zr (Al and Si) atoms, the frequency of exchange attempts is determined by Al, which is much less mobile in the titanium matrix compared to zirconium and silicon.

Within the framework of the approach used, the simulation time interval was determined by the relation *t = Pτ*. Here, *P* is the average number of jumps realized by an Al atom, and τ is the time required for a successful jump of an Al atom to a neighboring position, which can be expressed in terms of the diffusion coefficient of Al in titanium, *D_Al_ = d*^2^*/τ*, where *d* is the distance between the nearest neighbors in the α-Ti lattice.

To characterize the kinetics of the ordering that develops during the Monte Carlo simulation, we calculated the correlation function:

(3) $$S_{3}=P\left(Al_{3}|Al_{0}\right)=\frac{1}{Z_{3}c_{0}N}\sum_{i=1}^{N}\sum_{k=1}^{Z_{3}}n_{3k}\left(i\right)n_{0}{\left(i\right)}_{\;}$$

which determines the probability that two Al atoms are arranged relative to each other as third neighbors, i.e., in such coordination that is specific for the D0_19_ Ti_3_Al superstructure. Here, *Z_3_* is the coordination number for the third coordination sphere. The value of *S*_3_ can vary from *c*_0_ to 1. To describe the process of precipitation, the integral degree of decomposition *S_dec_* with respect to Al was calculated:

(4) $$S_{dec}=\frac{1}{2c_{0}\left(1-c_{0}\right)N}\sum_{i=1}^{N}{\left|c_{i}-c_{0}\right|}_{\;}$$

where *c_0_* is the average concentration of Al, *c_i_* is its local concentration obtained by averaging the occupation numbers over the neighborhood of the *i*-th site, and *N* is the number of lattice sites. The local concentration was calculated by averaging the occupation numbers of the given component over 6 coordination spheres:

(5) $$c_{i}=\frac{1}{Z_{sum}}\sum_{j=1}^{6}\sum_{k=1}^{Z\left(j\right)}n_{jk}\left(i\right),\;Z_{sum}=\sum_{j=1}^{6}Z\left(j\right),$$

where $n_{jk}\left(i\right)\;$ is the occupation number for the *k*-th site of the *j*-th coordination sphere around the *i*-th lattice site. The value of *S_dec_* is equal to zero in a completely disordered alloy and reaches a value of 1 with complete precipitation of pure Al. We should note that random fluctuations of the local concentration led to the fact that *S_dec_* differs remarkably from zero even in the initial state, where the atoms are arranged randomly. Therefore, the values of *S_dec_* presented below are for the time interval when the transient processes are completed, and the Al atoms are located mostly in the position of the third nearest neighbors relative to each other.

## 3. Results

**Figure 1:**
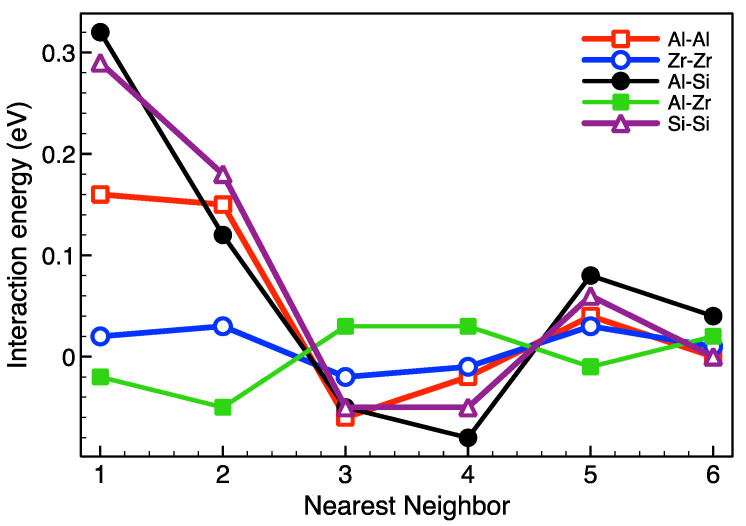
Energy of the effective pair interactions between alloying elements depending on their relative position in the hcp Ti lattice.

To study the processes of ordering and decomposition in Ti-based alloys, we used Monte Carlo simulation with first-principle parametrization of interatomic interactions (see Section 2). The energy of effective pair interactions (EPI) between the atoms of alloying elements in the matrix of hcp Ti, calculated by the PAW-VASP method, for lattice parameters a and c corresponding to the temperature T = 1000 K, is shown in Figure 1. Although the decrease in the lattice parameters with variation of temperature from 1000 K to 300 K is small, it leads to a significant increase (in absolute value) in the interaction energies; their change on the 3rd and 4th coordination sphere reaches 20%. Below, we use the interaction energies obtained for the lattice parameters corresponding to a temperature of 1000 K, which is close to the annealing temperatures used in [19,20,21].

**Figure 2:**
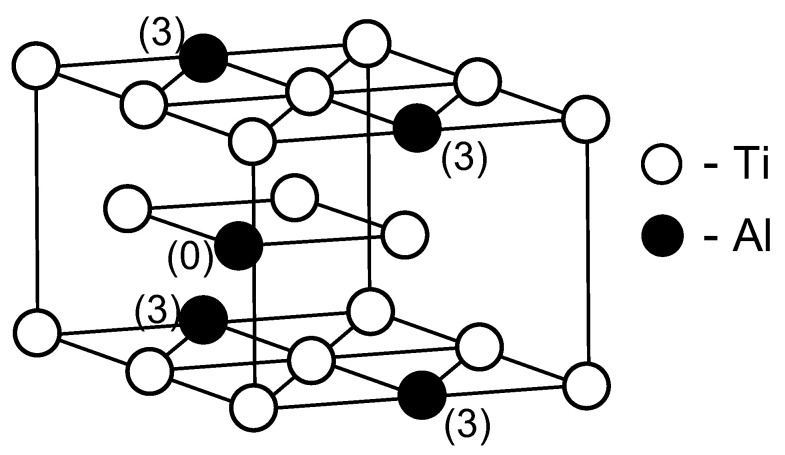
Unit cell of the Ti_3_Al alloy with the D0_19_ structure. The Al (3) atoms are in the position of third neighbors relative to the central Al (0) atom.

As can be seen from Figure 1, the arrangement of Al atoms in the positions of third neighbors relative to each other in the hcp Ti lattice is most energetically preferable. It is the arrangement of Al atoms that determines the energy gain during the formation of the DO_19_ superstructure in the Ti-Al alloy (Figure 2). The interaction energy between Zr atoms also has a minimum in the position of the third neighbors, but the corresponding value is much smaller than in the case of Al; indeed, there are no ordered phases in the phase diagram of Ti-Zr. The Al-Zr interaction is characterized by the energy gain of locating Zr atoms on the 1st and 2nd coordination sphere relative to Al, which is consistent with the preferential filling of the Ti sublattice by Zr atoms in the Ti-Al-Zr alloy [33]. As a result, one should expect a weak effect of Zr addition on Al ordering and the formation of Ti_3_Al precipitates in the Ti-Al-Zr alloy. In contrast with Zr, Si-Si and Al-Si interactions (Figure 1) were found to be quite similar to the Al-Al interactions. This is consistent with the broad field of coexistence of the Ti_3_Al and Ti_3_Si in the ternary phase diagram.

**Figure 3:**
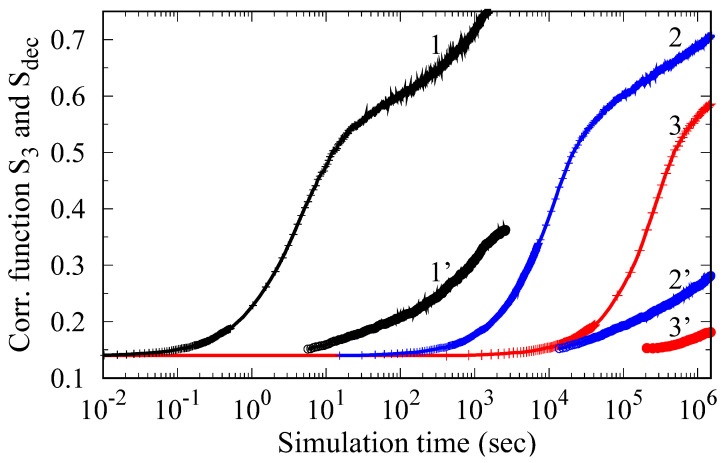
Ordering kinetics of the Ti-14%Al alloy obtained by Monte Carlo simulation at temperatures of 1000 K (curves 1, 1’), 850 K (curves 2, 2’) and 800 K (curves 3, 3’). Curves 1, 2, and 3 show the change in the correlation function *S_3_* over time, and curves 1’–3’ show the change in the parameter *S_dec_*.

The correlation function of third neighbors *S_3_* and the degree of decomposition *S_dec_* (Equations (3) and (4)) obtained from the Monte Carlo simulation of the phase transformation kinetics in the Ti-Al system are shown in Figure 3 depending on the annealing time. It can be seen that the transformation process develops in two distinct stages. First, there is a rapid partial ordering of the Al atoms, which is accompanied by an increase in *S_3_* to values around 0.5. At the next stage, corresponding to extended annealing times, diffusion of Al over long distances occurs, which results in the formation of ordered precipitates. In this case, the growth rate of *S_3_*(*t*) slows down, and the value of *S_dec_* increases.

**Figure 4:**
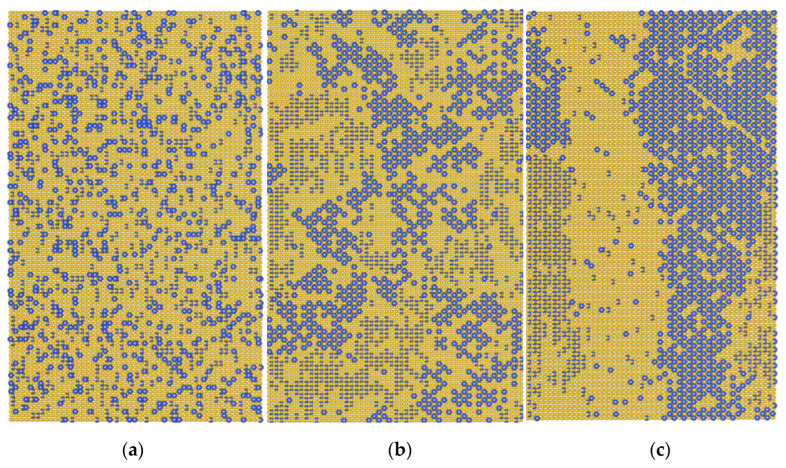
Distribution of Al atoms (blue circles) in the central section of the crystallite at different times in the Ti-14%Al alloy at temperature 850 K. (**a**) *t* = 0, (**b**) *t* = 7 × 10^4^ s, (**c**) *t* = 2.5 × 10^6^ s. Four (0110) layers are shown. Statistically uniform ordering (**b**) for the selected time is seen as alternating small antiphase domains.

The results presented in Figure 3 are consistent with the current conception [16,17,18,19,20,21,22] of the close relationship between the processes of ordering and the precipitation in the Ti-Al system. This behavior is determined by the peculiarities of the interaction of Al atoms in the Ti hcp lattice (Figure 1). In the first stage, a “uniform” ordering of Al atoms is realized (Figure 4b), while the energy gain is ensured by their arrangement in the position of third neighbors relative to each other. Note that in Figure 4b, which corresponds to the end of stage one, fine, ordered Ti_3_Al clusters become visible. With an increase in the exposure time, the formation of ordered precipitates occurs (Figure 4c), and there is a further decrease in energy because the fraction of third neighbors between Al atoms increases.

Another feature of the results presented in Figure 3 is that the incubation period and the duration of stage I of homogeneous ordering are very sensitive to temperature changes. So, if at 1000 K the total duration of the incubation period and stage I is about 10 s, then it increases to 10^4^ s when the temperature drops to 850 K. This dramatic change in kinetics is mainly due to a decrease in the diffusion rate of Al in Ti with decreasing temperature, and it should be considered when choosing the optimal regime of heat treatment of Ti-Al based alloys.

**Figure 5:**
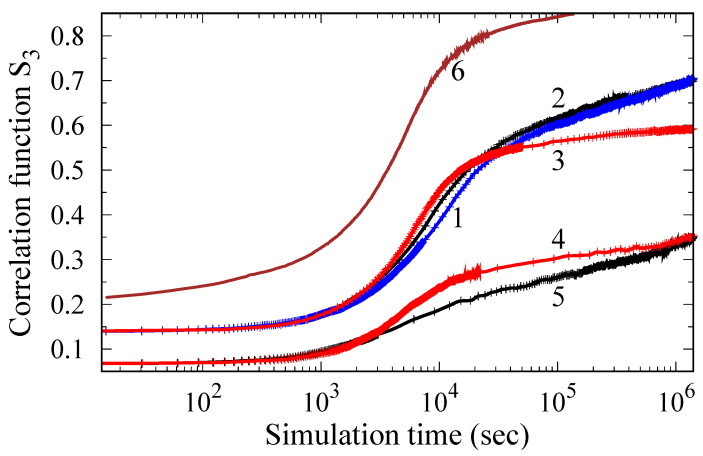
Ordering kinetics shown as the change in the correlation function *S_3_* over time for alloys Ti-14%Al (curve (1)), Ti-14%Al-7%Zr (2), Ti-14%Al-7%Si (3) and alloys Ti-7%Al-7%Si (4), Ti-7%Al-7%Zr (5) at 850 K. Curve (6) describes the variation *mS_3_* defined as the probability to find either the Al atom or the Si atom in the position of the third neighbor with respect to the chosen atom of Al.

In Figure 5, the results of Monte Carlo simulation of the ordering kinetics in Ti-Al alloys doped with Zr or Si for different solute atom concentrations are illustrated on a diagram. It can be seen that in alloys with 14% Al, the addition of Zr has little effect on the transformation kinetics; it is due to the features of the Al-Zr interaction (see Figure 1 and its discussion). A decrease in the Al content or substitution of a part of Al for Zr leads to a decrease in the degree of order and suppression of the first stage of alloy decomposition, which corresponds to homogeneous ordering (curve 5).

In the case of the addition of Si, which replaces Al atoms and promotes the formation of a superstructure D0_19_, the picture becomes more complex. The addition of Si leads to an acceleration of uniform ordering in stage I (curves 3, 4), but slows down the formation of third neighbors in stage II. This is because Al is replaced by Si atoms in the position of third neighbors, which results in a decrease in the correlation function *S_3_* determined by Equation (3). The modified correlation function *mS*_3_, defined as the probability P(Al_3_⋁Si_3_|Al_0_) when either the Al atom or the Si atom is in the position of the third neighbor with respect to the given aluminum atom, is described by curve 6. Comparing curves 3 and 6, we can conclude that the addition of Si will accelerate the ordering and formation of precipitates of the composition Ti_3_(Al_x_Si_1−x_). However, the actual picture of the development of decomposition processes in Ti-Al-Si alloys can be more complex (see Section 4).

Note that, in contrast to the magnitude of the correlation function S_3_, the start of ordering weakly depends on the composition of the alloy and is determined mainly by the temperature of annealing. This can be explained by the fact that the characteristic time when the ordering process begins is determined by the time of the diffusion jump of the slowest component of the alloy, in this case, Al.

## 4. Discussion

To study the kinetics of ordering in near-alpha titanium alloys, the atomistic simulations with ab initio interatomic interactions were employed. In agreement with experiments [6,9,16,17] and previous calculations [16], we have shown that the stage of homogeneous ordering precedes the formation of Ti_3_Al D0_19_ particles. In addition, we found that the duration of the homogeneous ordering is very sensitive to the annealing temperature and composition of alloy. In particular, the formation of Ti_3_Al precipitates starts after a few seconds at a temperature of 1000 K and requires about 5 × 10^5^ s at 800 K (Figure 3). The addition of Zr has little effect on the decomposition kinetics (curves 1 and 2 in Figure 5), while the Si addition, which occupies the Al sublattice, accelerates both the ordering and precipitation processes.

Our calculations predicted the formation of the D0_19_ phase of composition Ti_3_(Al_x_Si_1−x_), curve 6 in Figure 5. However, such a phase is absent in the equilibrium phase diagram of the ternary Ti-Al-Si alloy [34], although the phases Ti_3_Si and Ti_5_Si_3_ are present in the binary diagram. The complex phase Ti_3_(Al_x_Si_1−x_) is apparently a transition state that can transform into phases Ti_3_Al, Ti_3_Si, and Ti_5_Si_3_ at rather long simulation times. Therefore, we assume that our results correctly describe the early stages of precipitation.

Generally speaking, many-body interactions and vibrational entropy make a significant contribution to the phase equilibrium in the Ti-Al system [35,36]. Nevertheless, we believe that considering pair interactions is sufficient for a correct description of the main features of the decomposition kinetics in system Ti-Al-X.

**Figure 6:**
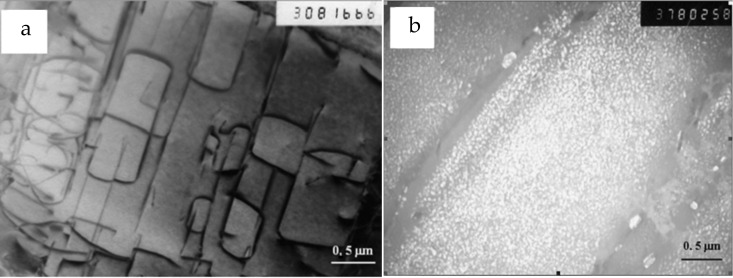
Microstructures of alloys after aging at (**a**) 500 °C (TEM bright-field image) and after aging at (**b**) 700 °C (dark-field image) [19].

The features discussed here of the ordering kinetics in the Ti-Al-X system open a possibility of controlling the structural state of these alloys. As shown for complex-alloyed system Ti-Al-Zr-Sn [19], an increase in the annealing temperature leads to a change in the mechanism of decomposition and α + α_2_ microstructure formation. In an alloy with 12at% Al at temperatures below 600 °C, uniform ordering occurs during long-term holding; in this case, antiphase boundaries are observed in the structure, and the domain sizes reach 300–400 nm (Figure 6a). At the same time, annealing at 700 °C leads to the appearance of many dispersed ordered particles (Figure 6b), which are relatively evenly distributed over the grains, and their size after 100 h of holding does not exceed 50 nm, wherein fracture toughness undergoes a remarkable decrease [23]. This observation agrees with results of our calculations presented in Figure 3, where ordered precipitates are formed in relatively short times at 1000 K and ordering stage I becomes very long (about 100 h) when the annealing temperature drops to 800 K. In this case, a set of antiphase domains is formed in the structure during Monte Carlo simulation (Figure 4b).

Our investigation [19] of phase transformation in the system Ti-17%Al-X (Zr, Sn in the presence of Mo and Nb) showed that if Sn initiates the process of intermetallic formation, then zirconium, at least, does not accelerate it. The results of our Monte Carlo simulations for Ti-14%Al-7%Zr alloy (see Figure 5) are consistent with the last conclusion and predict an acceleration of the ordering process in Ti-Al with the addition of Si.

As we have mentioned above, the formation of the precipitates of Ti_3_Al at high temperatures can occur very quickly (in a few seconds at 1000 K, see Figure 3). Therefore, it is not surprising that after quenching the Ti-17 at.% Al alloy from a temperature of 950 °C (which corresponds to single-phase α-region), homogeneously distributed dispersed precipitates of Ti_3_Al with a size of up to 10 nm were observed in [20]. During subsequent and rather long annealing at temperatures of 500–700 °C, certain growth of these regions up to sizes of about 40 nm was observed, while they remained coherent with the matrix.

**Figure 7:**
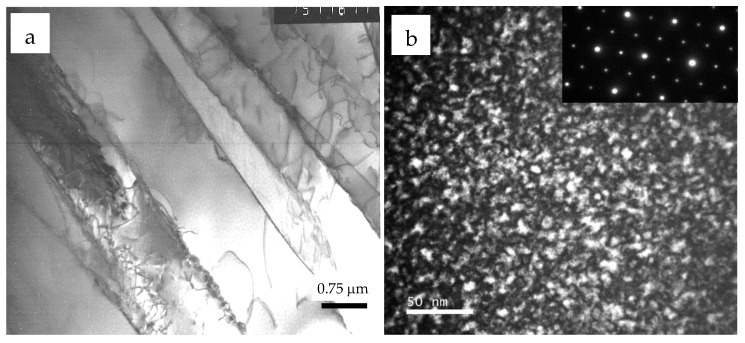
Microstructure of the Ti-17 at.% Al alloy after quenching from 1200 °C (β-region) (**a**) and annealing at 700 °C for 100 h (**b**) [20].

When we were performing Monte Carlo simulations, the processes of heterogeneous nucleation of a new phase were neglected. As shown in [20,37], the heterogeneous nucleation at internal interfaces can significantly change the transformation kinetics. The increase in the heating temperature to 1200 °C (β-region) and subsequent quenching leads to the realization of the martensitic β → α’ transformation; martensite plates are observed in the structure (Figure 7a), and reflections from the α_2_ phase are not detected. Subsequent annealing at temperature 550–700 °C leads to the decomposition of α’ martensite and the precipitation of dispersed particles of the Ti_3_Al intermetallic (Figure 7b). This result indicates that the creation of a significant density of defects during the martensitic transformation provides a number of nucleation centers and a new phase is formed through subsequent annealing by the nucleation and growth mechanism. In this case, the stage I of homogeneous ordering can be suppressed.

## 5. Conclusions

The ordering and decomposition kinetics in Ti-Al-X (X = Si, Zr) alloys were studied using Monte Carlo simulations with ab initio interatomic interactions. The simulation results are in good agreement with experimental data on the transformation kinetics and allow explaining the features of microstructure formation in near-α Ti-Al based alloys.

(1) It was shown that the processes of ordering and precipitation in the Ti-Al system are closely related, and homogeneous ordering precedes the precipitation of Ti_3_Al particles. This result is consistent with the widespread concept [16,17,20,21,22] that the transformation in near-α Ti-Al based alloys develops according to the conditional spinodal mechanism.
(2) It was found that the characteristic time of the beginning of ordering is determined by the rate of diffusion of Al, which is the slowest component of the alloy, and the duration of homogeneous ordering is very sensitive to the annealing temperature and alloy composition.
(3) For a given annealing time, uniform ordering can be replaced by the precipitation with increasing temperature. This opens the possibility of controlling the structural state of the alloy by choosing the heat treatment regime and chemical composition.

It should be noted that the approach used does not take into account the possibility of heterogeneous nucleation of α_2_ phase. The presence of heterogeneous nucleation centers can suppress the stage of uniform ordering; this issue requires further consideration.

## Data Availability

Not applicable.
